# Biomarkers to quantify cell migration characteristics

**DOI:** 10.1186/s12935-020-01312-w

**Published:** 2020-06-05

**Authors:** Sangwoo Kwon, Woochul Yang, Donggerami Moon, Kyung Sook Kim

**Affiliations:** 1 Department of Biomedical Engineering, College of Medicine, Kyung Hee University, 1 Hoegi-dong, Dongdaemun-gu, Seoul, 04620 Republic of Korea; 2 Department of Physics, Dongguk University, Seoul, 100-715 Republic of Korea

**Keywords:** Cell movement, Cellular elasticity, Adhesion strength, F-actin, Integrin

## Abstract

**Background:**

Because cell movement is primarily driven by the connection between F-actin and integrin through a physical linkage, cellular elasticity and adhesion strength have been considered as biomarkers of cell motility. However, a consistent set of biomarkers that indicate the potential for cell motility is still lacking.

**Methods:**

In this work, we characterize a phenotype of cell migration in terms of cellular elasticity and adhesion strength, which reveals the interdependence of subcellular systems that mediate optimal cell migration.

**Results:**

Stiff cells weakly adhered to the substrate revealed superior motility, while soft cell migration with strong adhesion was relatively inhibited. The spatial distribution and amount of F-actin and integrin were highly variable depending on cell type, but their density exhibited linear correlations with cellular elasticity and adhesion strength, respectively.

**Conclusions:**

The densities of F-actin and integrin exhibited linear correlations with cellular elasticity and adhesion strength, respectively, therefore, they can be considered as biomarkers to quantify cell migration characteristics.

## Background

Cell movement is a fundamental cellular function for establishing and maintaining proper organization. The movement of cells is closely related to important biological functions such as wound healing, immune response, angiogenesis, and cancer metastasis [1–3]. Cell movement can be categorized by motility and migration. Motility refers to spontaneous non-directional movement, while migration is directional movement in response to a cell attractant or repellent.

Cell migration is orchestrated by cytoskeletal change and the formation of focal adhesion. Cell migration involves the following process: (i) protrusion of the leading edge, (ii) formation of focal adhesion at the leading edge and detachment at the trailing edge, and (iii) movement of the cell body [4, 5]. During migration, actin is polymerized at the protrusion of the leading edge and depolymerized at the trailing edge repeatedly. The polymerization and bundling of F-actin causes stiffening of the cells, while depolymerization makes the cells soft [6]. After actin is polymerized at the protrusion, adhesions are assembled near the leading edge. The adhesions are maturated by the dynamic cross-linking of F-actin. The adhesions then disassemble at the trailing edge when the linkage between the F-actin and integrin is broken. The physical interaction between the F-actin and integrin provides the traction force for cell migration.

Cell migration has long been a scientific subject of interest, not only in basic research but also in practice. Because cancer metastasis associated with cell mobility remains the greatest challenge in cancer treatment, numerous studies have been conducted to understand motility from the viewpoint of clinical management. The development of cellular elasticity measurement technology, such as atomic force microscopy (AFM) and micropipette aspiration, reveals that the elasticity and mobility of cancer cells are correlated, whereby elasticity has been recognized as a biomarker of the invasive potential of cancer cells [7, 8]. Several studies have demonstrated the relation between the elasticity and metastatic potential of the cells; however, some reports are inconsistent with others. For example, highly metastatic ovarian cancer cells (HEY A8) are softer than non-malignant ovarian epithelial cells, and the invasiveness of HEY A8 is associated with actin cytoskeleton remodeling [9]. Conversely, stiff breast cancer cells (MDA-MB-231) have exhibited excellent migratory behavior in dense culture conditions [10]. The characteristics of adhesion to the extracellular matrix (ECM) in cancer cells are different from those of normal cells, and are also correlated with the invasive and metastatic potential of cancer cells [11]. Adhesion strength is generally reduced in cancer cells, and the alterations depend on the cell type and oncogene [12, 13]. Adhesion strength is heterogeneous in metastatic cells under stromal-like conditions due to their increased sensitivity to Mg^2+^ and Ca^2+^ mediated focal adhesion disassembly [14]. If the cells are strongly adherent to the ECM, their migration is inhibited.

Although there has been much progress in understanding cell motility, a consistent set of biomarkers to quantify cell migration characteristics is still lacking. Therefore, in this work, we focus on identifying factors that affect cell movement, and analyze their contributions qualitatively and quantitatively. The contributions of cellular elasticity and adhesion to cell movement were investigated in one normal breast cell (MCF10A) and three breast cancer cells (MCF7, T47D, and MDA-MB-231). The differences in cellular elasticity between the cells are explained in terms of the amount and distribution of F-actin. The adhesion strength between the cells and the ECM is explained in terms of the amount and distribution of integrin.

## Materials and methods

### Cell culture

Three kinds of breast cancer cells including MCF7, T47D, and MDA-MB-231 were purchased from Korean Cell Line Bank (KCLB, Seoul, Korea). Three cancer cells were cultured in Roswell Park Memorial Institute 1640 medium (RPMI) (Thermo Fisher Scientific Inc., MA, USA) with 10% FBS, 1% antibiotics/antimycotics, 300 mg/l l-glutamine, 25 mM hydroxyethyl piperazineethanesulfonic acid (HEPES), and 25 mM NaHCO_3_. The normal breast cell (MCF10A) was purchased from ATTC (ATCC Inc., Virginia, USA). The culture medium of MCF10A included Dulbecco’s Modified Eagle Medium/nutrient mixture F-12 (DMEM/F-12) (Thermo Fisher Scientific Inc., MA, USA), 5% horse serum, 20 ng/ml EGF, 0.5 mg/ml hydrocortisone, 100 ng/ml cholera toxin, 10 µg/ml insulin, and 1% antibiotics/antimycotics.

### 2-Dimensional (2D) optical tracking assay

Cells were cultured at a low density of 0.5 × 10^4^ cells/cm^2^ in a Petri dish (35 × 10 mm^2^) for real-time observation of the 2D cell motility. A portable incubator (Chamlide TC; CU-501, Live Cell Instrument Inc., Seoul, Korea) was employed for long-term observation (8 h). The cell motility was measured at 1 min intervals based on the location of the nucleus. The optical images that tracked the movement of the cells were analyzed as 2D scalar values using a video analysis program (Tracker, Video analysis and modeling tool, Softmedia).

### Three-dimensional (3D) migration assay

Cell migration ability was analyzed utilizing polycarbonate membrane inserts with an 8 µm pore size (CytoSelect™ 24-well cell migration assay, Cell Biolabs, Inc. CA, USA). Serum-free media were placed on top of the polycarbonate membrane, and culture media were placed under the membrane. Cells of 4.62 × 10^5^ were injected into the serum-free media, and then incubated for 8 h. The cells remaining on the serum-free media, and the cells that had migrated to the culture media were stained for 10 min for optical observation. The migrated cells were counted and analyzed using an MTT assay.

### AFM and force–distance (FD) curve measurement

The elastic property of cells was measured by AFM (Nano N8 Neos, Bruker^®^, Germany) in liquid conditions. The FD curve was measured in contact mode with an Au-coated probe (ContGD, BudgetSensors Inc. Sofia, Bulgaria) to enhance the degree of laser deflection from the cantilever to the photodetector for signal detection. The detailed dimensions of the probe were as follows: resonance frequency of 13 kHz (± 4 kHz), force constant of 0.2 N/m (0.07–0.4 N/m), cantilever length of 450 μm (± 10 μm), cantilever width of 50 μm (± 5 μm), cantilever thickness of 2 μm (± 1 μm), tip height of 17 μm (± 2 μm), and tip radius of < 10 nm. The load force was set to 10 nN or less to minimize damage to the cell membrane, and the loading rate of the probe was approximately 1 µm/s. The FD curve was measured around the cell nucleus to avoid matrix effects at 10 different regions per cell. About 20 cells were considered for each cell type. Considering a relatively sharp probe with a radius of approximately 10 nm, the FD curve was analyzed using the Sneddon model, which is as follows [15].

1 $$ {\text{F}} = {\text{E}} \times \left[ {\frac{2\tan \alpha }{{\pi \times \left( {1 - v^{2} } \right)}}} \right] \times \delta^{2} $$

where F and *δ* are the load force and the indentation depth, respectively. *α* is the half-cone angle along the cantilever axis, which was 22.5° in this experiment. *ν* is the Poisson’s ratio, which was assumed to be 0.5.

### Immunofluorescence staining

Cells were fixed with a 3.7% formaldehyde solution for 15 min and washed with a phosphate buffer saline (PBS) solution for 30 s. Rhodamine-phalloidin (100 nM, Alexa Fluor^®^ 488 phalloidin, Invitrogen Inc., CA, USA) was used for detecting F-actin. The reagent-treated cells were incubated in the dark at room temperature for 30 min, and then re-washed several times with PBS and stored in the dark at 4 °C. For integrin fluorescence staining, the cells were permeabilized in 0.5% TritonX/PBS for 5 min and blocked with bovine serum albumin (BSA) (GenDEPOT Inc., Texas, USA) for 30 min at 21 °C. The cells were then incubated with antibody (Cat. No. 24693, 1/200, Abcam Inc., Cambridge, UK) for 1 h at 21 °C. The secondary antibody of Alexa Fluor^®^ 555 goat anti-mouse IgG (H + L) (Invitrogen Inc., CA, USA) was used at a 1/500 dilution for 1 h at 21 °C. A fluorescence image was detected using the fluorescence optical microscope (NIKON Ti-E, Nikon Instruments Inc., Tokyo, Japan). Rhodamine-phalloidin is a green fluorescence reagent with an excitation of approximately 495 nm and emission at approximately 518 nm. Alexa Fluor^®^ 555 is an orange fluorescence reagent with an excitation of approximately 555 nm and emission at approximately 565 nm.

### Western blotting

To determine the content of F-actin, cells were washed several times with PBS, and then scraped in a RIPA buffer containing a protease inhibitor cocktail. For the separation of actin proteins, cell debris was centrifuged at 374×*g* for 5 min at 4 °C. The supernatant was centrifuged at 15,000×*g* for 5 min at 4 °C. F-actin in pallet form was separated, and G-actin was present in the remaining solution. Briefly, 60 µg of G- or F-actin proteins were loaded in 12.5% polyacrylamide gels, and the resolved proteins were transferred to nitrocellulose membranes. The transferred proteins were blocked with 5% fat-free milk in PBS (pH 7.4) for 30 min at room temperature, and then incubated with anti-actin (Cytoskeleton, Denver, CO, USA)/Tris buffered saline with Tween^®^ 20 (TBS-T) at a 1/500 dilution overnight at 4 °C. Finally, the membranes were incubated with anti-rabbit secondary antibodies/fat-free milk at a 1/6500 dilution for 1 h at room temperature.

Integrin was analyzed using a similar process. The process, briefly, is as follows. The lysates were incubated for 30 min at 4 °C and then centrifuged for 20 min at 12,000 rpm. The supernatant was mixed with an equal amount of loading buffer (2 × Laemmli sample buffer with 5% beta-mercaptoethanol) and boiled for 5 min. The size marker (6 µl) and protein (40 µl) were separately loaded in 8.0% polyacrylamide gels. The resolved proteins were transferred to nitrocellulose membranes, blocked with 5% BSA/TBS-T for 1 h at room temperature, and then incubated with a primary antibody (anti-integrin beta 1 antibody [P5D2], Abcam Inc., Cambridge, UK) at a 1/1000 dilution overnight at 4 °C. The secondary antibody (Alexa Fluor^®^ 555 goat anti-mouse IgG (H + L), Cambridge, UK) was incubated with blocking buffer at a 1/5000 dilution for 1 h at room temperature. Finally, the membranes were subjected to enhanced chemiluminescence (Pierce Biotechnology, MA, USA) and autoradiography using the ChemiDoc XRS + Imaging System (BioRad, Hercules, CA, USA).

### Disruption of F-actin and integrin

Cells were cultured at a low density of 0.5 × 10^4^ cells/cm^2^ in a Petri dish for optical observation. To study the effect of disruption of F-actin and integrin on cellular mechanics, the cells were treated with latrunculin A (LatA) (500 nM) and trypsin–EDTA (0.05% W/V), separately. The morphological change in cells induced by LatA and trypsin–EDTA was observed in real time.

### Adhesion strength

The adhesion strength between the cells and substrate was measured with the spinning disk technique. Cells were seeded at a density of 6.7 × 10^4^ cells per culture dish (60 × 15 mm^2^) with media, and the culture dish was mounted on a spinning disk. The disk was rotated at 3000 rpm utilizing a spin process controller (MIDAS^®^, Daejeon, Korea). The fraction of adherent cells was quantified by counting the number of cells before and after spinning by using optical microscopy images.

### Statistics

All data were represented as mean ± SEM (standard error of the mean). The statistical analyses were performed based on a two-tailed Student’s t-test. P-values less than 0.05 were regarded as statistically significant. Note that *p ≤ 0.05, **p ≤ 0.01, ***p ≤ 0.001, ****p ≤ 0.0001.

## Results

### Cell movement in 2D and 3D conditions, and cellular elasticity

A wound healing assay is frequently utilized in cell movement observation. A wound gap in a cell monolayer is created by scratching, and then cell migration toward the gap is monitored using time lapse microscopy [16–19]. The wound healing assay is an accessible and useful method specifically for collective cell migration.

**Fig. 1:**
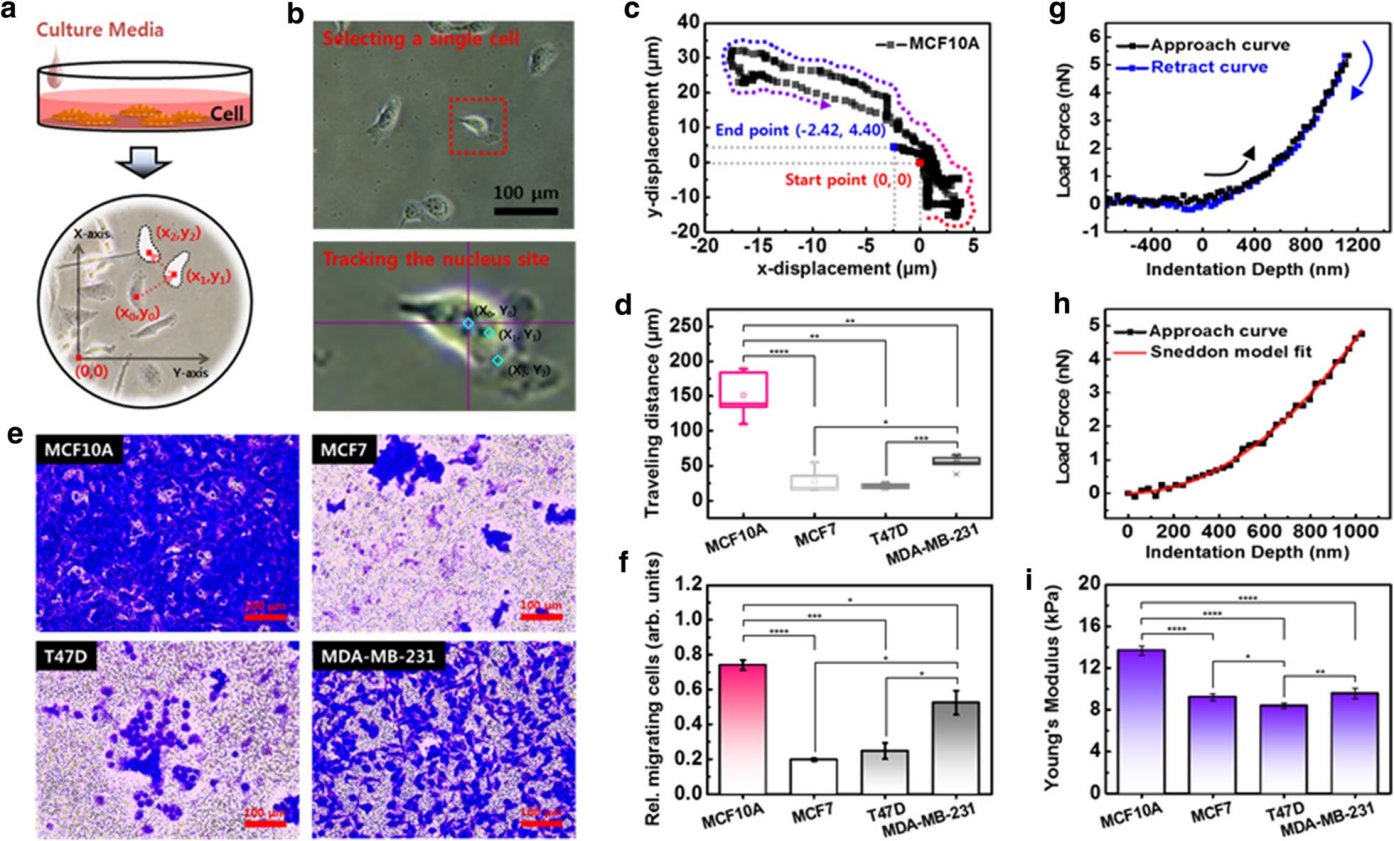
Cell movement in 2D and 3D conditions, and cellular elasticity. **a** Schematic diagram of a single cell tracking system using an optical microscope. Two-dimensional cell movement was tracked in Cartesian coordinates at 1 min intervals for 8 h. **b** At each time point, the cell location was determined based on the nucleus. **c** Cell displacement was converted to a scalar quantity. **d** Comparison between cells of traveling distance on a 2D substrate. **e** Three-dimensional migration ability was evaluated with a transwell membrane assay. The cells migrated through an 8 µm pore were stained in blue. **f** Relative number of migrated cells. **g** Representative FD curve of the deformable cells. The approach and retraction curve are marked with black and blue rectangular points, respectively. **h** Approach curve was fitted to the Sneddon model to obtain the Young’s modulus of the cell. **i** Determined Young’s modulus of the cells. Each value represents mean ± SEM (*p ≤ 0.05, **p ≤ 0.01, ***p ≤ 0.001, ****p ≤ 0.0001)

However, cells do not move linearly in one direction, but move in all directions haphazardly. Therefore, the direction of cell movement should be considered as a vector for quantitative analysis. Therefore, we designed a single cell tracking system (SCTS) based on optical microscopy to evaluate the movement of a single cell unit. We randomly selected 10 cells in each group, and their movements in a 2D substrate were traced individually. The cell movement path was measured by scalar values along the *x* and *y*-axis at 1 min intervals for 8 h (Fig. 1a). The location of the cells at each point was determined based on the nucleus, which is less varied in position and morphology and can be seen more clearly than the membranes (Fig. 1b). Only cells with one nucleus were considered in tracking (Additional file 1: Fig. S1). One cell traveled on a path described by Cartesian coordinates from (0, 0) to (−2.42, 4.40) over 8 h (Fig. 1b). The distance of movement of a single cell was converted to a scalar quantity at each time point, and then the total distance was added. There was a large difference between the normal cells and the cancer cells in the distance traveled over 8 h. The normal MCF10A cells traveled 151.25 ± 15.22 μm over 8 h; however, the three types of cancer cells moved less than half of the traveling distance of MCF10A (Fig. 1d). There was also a difference in the traveling distance between cancer cells. The metastatic cancer cells MDA-MB-231 moved 54.71 ± 4.70 μm; however, the two non-metastatic cells MCF7 and T47D moved a relatively short distance of 28.13 ± 7.71 μm and 21.06 ± 1.68 μm, respectively.

To analyze the directional movement of single cells in three dimensions (3D), we employed the transwell migration assay. The transwell assay examines the ability for cell migration toward a chemo-attractant by the insertion of a small pore, which was fixed at 8 µm in this work. The same density of cells was placed above the insertion (serum-free medium) in all groups, and the number of cells migrated through the pore (culture medium) was analyzed after 8 h incubation. The migrated cells were stained in blue (Fig. 1e). It was apparent that most of the MCF10A had migrated through the pore, while the percentage of the three cancer cells that had migrated was considerably lower. By analyzing the absorbance intensity of the stained area, the migration ability of the cells was quantitatively compared (Fig. 1f). The absorbance intensity of MCF10A was 0.74 ± 0.03, which was the largest value. Of the cancer cells, the MDA-MB-231 showed the largest value of 0.53 ± 0.07, followed by T47D (0.25 ± 0.05) and MCF7 (0.20 ± 0.01). This result shows good agreement with the results of cell movement in 2D. In the collective movement of cells, the migration depends on the cell density [10]. Since our purpose was to compare the mobility of four cells relatively, the experiment was conducted at the same density of 4.62 × 10^5^/ml. The rate of movement of each cell is expected to vary with cell density, but the differences between cells may not change significantly.

Cellular elasticity can be determined by AFM at the nano/micro scales [20]. An AFM detects the change in force between a sample surface and the AFM tip, which is called the FD curve, at the atomic level. This change was converted to the Young’s modulus of the sample based on a theoretical model [21]. The FD curve is composed of approach and retraction curves, and the curves are mostly nonlinear in deformable biological materials (Fig. 1g). The FD curve was measured at 10 different locations on a cell, specifically on the cell body to avoid the effects of the nucleus and substrate. Approximately 20 different cells were measured in each group. The Young’s modulus of the cells was extracted from the approach curve based on the Sneddon model (Fig. 1h) [15]. The indentation depth of each cell was approximately 1000 nm for a loading force of 5 nN. The determined Young’s moduli were as follows: MCF10A (13.69 ± 0.44 kPa), MCF7 (9.24 ± 0.32 kPa), T47D (8.39 ± 0.29 kPa), and MDA-MB-231 (9.57 ± 0.50 kPa) (Fig. 1i). The result indicates that the normal cells were 1.43–1.63 times stiffer than the cancer cells. The differences between the cancer cells were statistically significant.

### Comparison of F-actin distributions and content, and depolymerization effects of F-actin on cell morphology

**Fig. 2:**
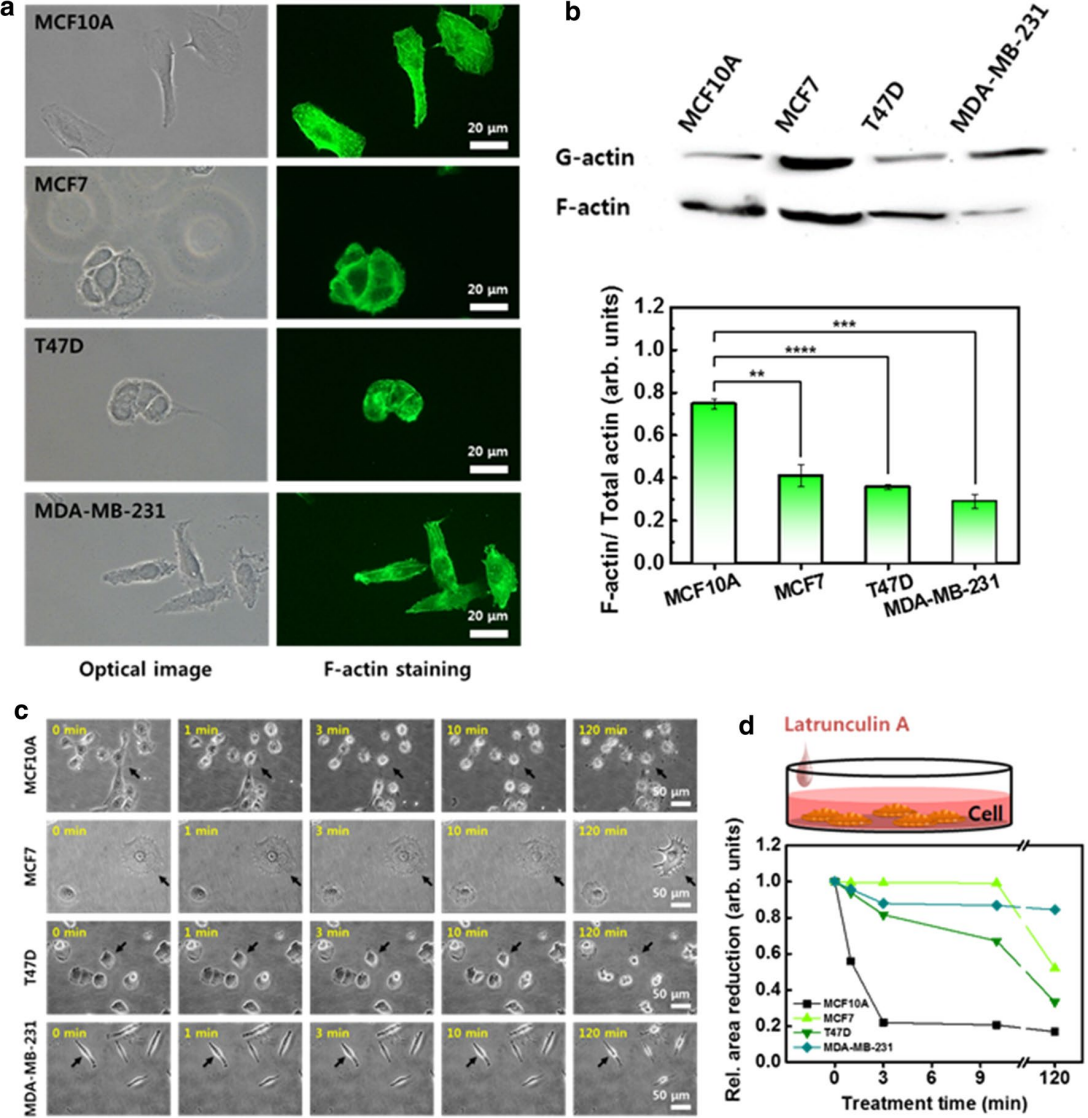
F-actin distribution, amounts, and depolymerization. **a** Distribution of F-actin in four different cells, observed by optical (left column) and fluorescence (right column) microscopy. **b** Western blot analysis of soluble actin (G-actin) and insoluble actin (F-actin). The ratio of F-actin to total actin (G- and F-actin). **c** Morphological change in cells induced by the LatA treatment. The same position on the cell was indicated with an arrow to compare morphological changes according to the treatment time. **d** Relative cell area reduction as a function of LatA treatment time. Each value represents mean ± SEM (*p ≤ 0.05, **p ≤ 0.01, ***p ≤ 0.001, ****p ≤ 0.0001)

Actin is a major constituent of the cytoskeleton and plays a crucial role in both cell motility and elasticity. Actin has two forms: free monomer (G-actin), and microfilament (F- actin). G-actin polymerizes into F-actin under appropriate conditions, and F-actin depolymerizes into G-actin. Cell movement is generally initiated by polarization of the cell morphology through F-actin alignment. When a cell moves, the F-actin organizes high-order bundles at the leading edge of the cell, such as filopodia and lamellipodia, and aligns them in the direction of migration. The F-actin bundles exert traction forces for movement, and the stressed bundles cause cell stiffening. In MCF10A, the F-actin was distributed throughout the whole cytoplasm, and the actin bundles were densely aligned in all directions with well-defined stress fibers (Fig. 2a). In contrast, the F-actin were less organized and were distributed along the edge of the cell membrane in the three cancer cells.

F-actin content is closely related to cellular elasticity, even though it continually fluctuates to some extent due to changes in internal or external environmental conditions. Therefore, the soluble and insoluble fractions containing G- and F-actin were analyzed by western blot assay, and the result was expressed as the ratio of F-actin to total actin (G- and F-actin) (Fig. 2b). The MCF10A showed the highest values for the F-actin ratio, which was 0.75 ± 0.02, among the cells considered. The three cancer cells showed significantly lower values for F-actin ratio than the normal cells. The value for MCF7 was 0.41 ± 0.05, followed by T47D at 0.36 ± 0.01 and MDA-MB-231 at 0.29 ± 0.03, respectively.

Cell movement requires both the polymerization and depolymerization of actin, depending on the traveling direction. Therefore, it is meaningful to compare the depolymerization rate of F-actin and the cell morphological changes induced by the depolymerization between cells. The cells were treated with LatA reagent to disrupt the F-actin, and the induced changes were observed for 120 min (Fig. 2c). To compare the contraction according to the LatA treatment time, the same position was marked in each optical image with a black arrow. The MCF10A showed a very rapid response to the LatA treatment, indicating sensitivity to the depolymerization of F-actin. The morphology changed after 1 min, and most cells were floating after a 10 min treatment. In the cancer cells, no significant changes were observed for a relatively long LatA treatment of 10 min. A noticeable decrease in cell area by depolymerization was observed within 3 min in all cells (Fig. 2d). The cell area reduction rate within 3 min was the largest for MCF10A (77.9%), followed by T47D (18.3%), MDA-MB-231 (12.1%), and MCF7 (0.7%).

### Comparison of integrin-based adhesion properties of cells

**Fig. 3:**
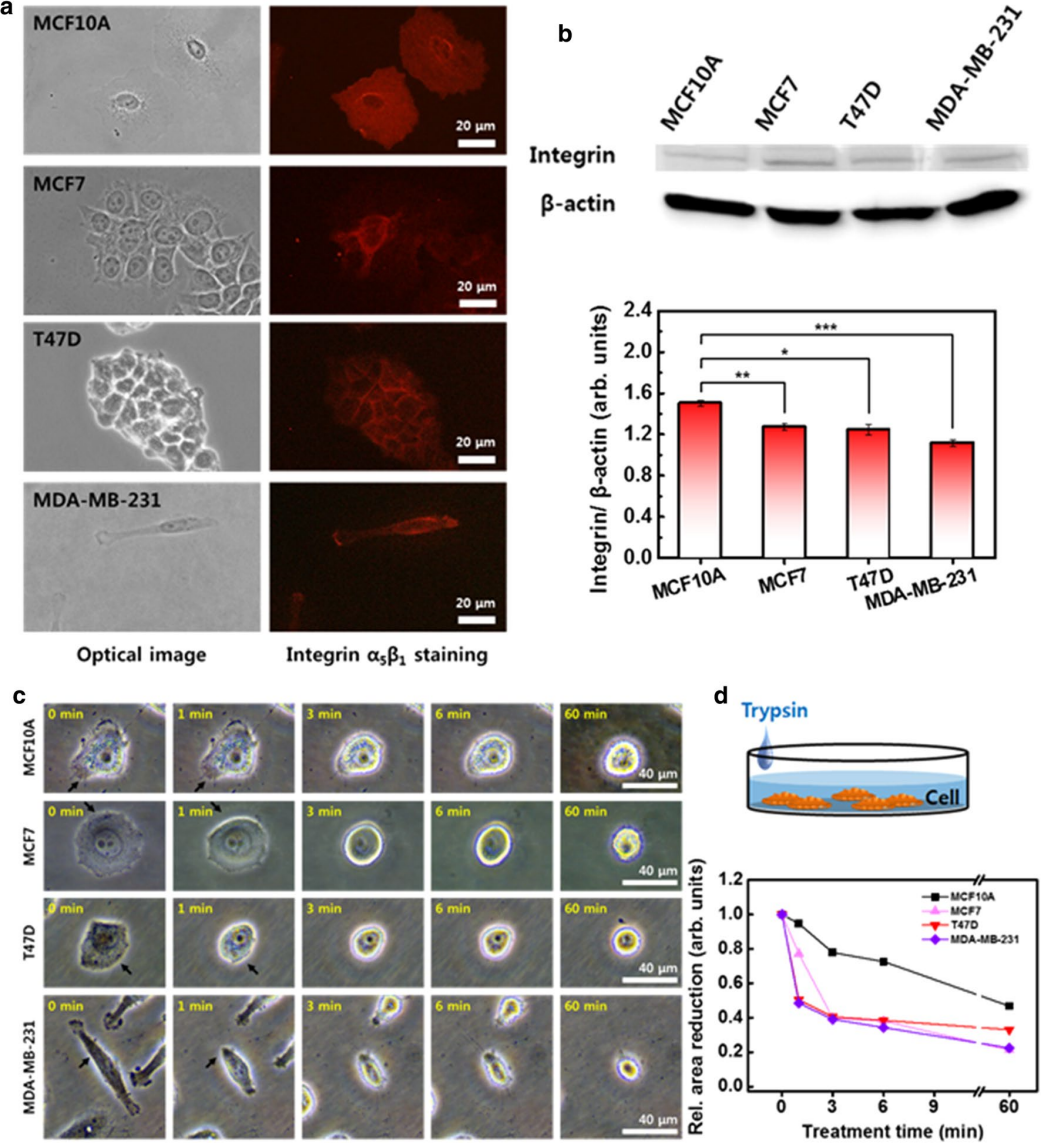
Integrin distribution, amounts, and disassembly. **a** Distribution of integrin in four different cells observed by optical (left column) and fluorescence (right column) microscopy. **b** Western blot analysis of integrin, and the ratio of integrin to β-actin in four cells. **c** Morphological change of cells induced by trypsin–EDTA treatment. The same position on the cell was indicated with an arrow to compare the morphological change according to the treatment time. **d** Relative cell area reduction as a function of trypsin–EDTA treatment time. Each value represents mean ± SEM (*p ≤ 0.05, **p ≤ 0.01, ***p ≤ 0.001, ****p ≤ 0.0001)

Cells attach to their substratum through specialized protein complexes, and cell movement requires dynamic interaction between a cell and the substratum. Integrin is the major transmembrane receptor that facilitates adhesion between the cell and the ECM. In addition, integrin mediates the dynamic interactions between the intracellular F-actin and the ECM during cell movement. To discuss the effects of integrin on cell movement, the integrin distributions and content were measured and compared between the cells. The integrin was distributed similar to F-actin. The integrin was distributed throughout the whole cytoplasm in the normal MCF10A cells, while in the in the MCF7, T47D, and MDA-MB-231 cancer cells, it was mainly located along the periphery region (Fig. 3a). The MCF10A contained a relatively larger content of integrin than the cancer cells. The relative integrin content of MCF10A was 1.51 ± 0.03, followed by MCF7 at 1.27 ± 0.03, T47D at 1.25 ± 0.05, and MDA-MB-231 at 1.12 ± 0.03 (Fig. 3b).

Integrin-mediated adhesions dynamically form and turn over during cell migration. After the protrusions are formed toward the direction of migration, the integrin physically contacts with the ECM. The F-actin links to the ECM through the contacts, which are referred to as focal adhesions, and exerts a traction force to push the plasma membrane forward. The integrin must be in a high-affinity state with the ECM at the leading edge [22, 23]. The focal adhesion slides in the direction of migration, which causes a reduction in focal contact size and dispersion at the trailing cell rear. Cells detach from the trailing edge through disassembly of the adhesion, and the rear retraction rate controls the speed of migration depending on the adhesiveness. To compare the adhesiveness, the integrin-mediated adhesion was disrupted by trypsin–EDTA, and the detachment process was monitored for 60 min (Fig. 3c). The reaction of cells to the adhesion disassembly was quite different from that of F-actin depolymerization. In a short treatment of 1 min, MCF10A showed a slight change in morphology, while the morphology of MCF7, T47D, and MDA-MB-231 was significantly shrunk, as indicated by arrows (Fig. 3c). The reduction in cell area by trypsin–EDTA was also significant in the three cancer cells (Fig. 3d). The area of MCF10A gradually decreased.

### Adhesion strength between cells and ECM

**Fig. 4:**
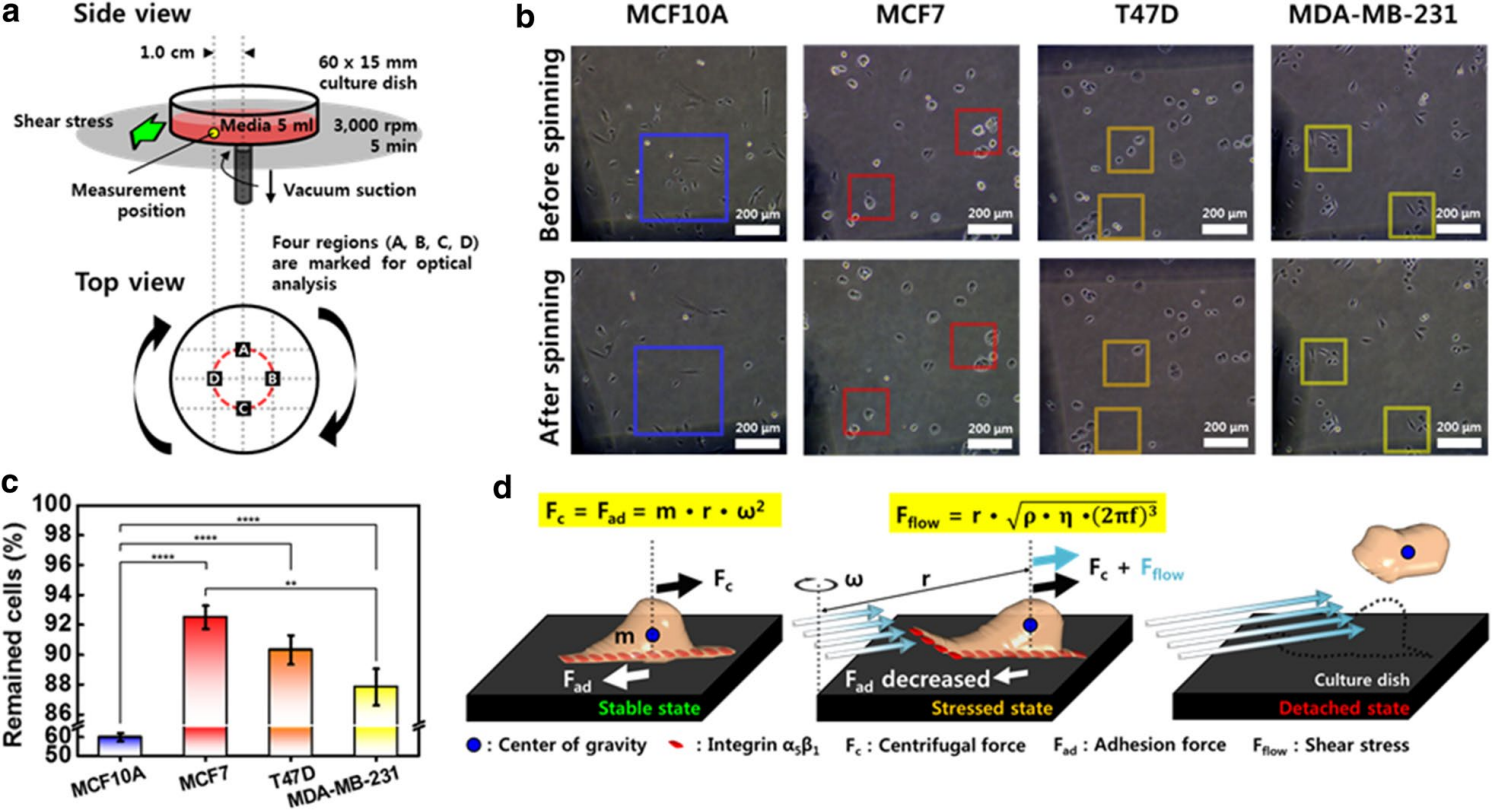
Physical adhesion strength test utilizing spinning disk technique. **a** Schematic diagram of spinning disk system. **b** Optical images of cells before and after the spinning. The number of attached cells before and after the spinning was compared at the same position indicated by a square. **c** Comparison of remaining cells after the spinning. **d** Model of forces acting on cells and their interaction. Each value represents mean ± SEM (*p ≤ 0.05, **p ≤ 0.01, ***p ≤ 0.001, ****p ≤ 0.0001)

The spinning disk technique was utilized to determine the adhesion strength between the cells and the substrate. Cells in medium were rotated at a speed of 3000 rpm for 5 min. Because the shear stress exerted by the rotating medium depends on the position, the fraction of adherent cells were quantified at the same position from the center (Fig. 4a). Four different regions located 1.0 cm away from the center of rotation were marked, and the number of cells was counted before and after the spinning. The shear stress (*τ*) by the radial motion of the medium over the surface of the culture dish can be calculated as follows.

2 $$ \tau = r\sqrt {\rho \eta \left( {2\pi f} \right)^{3} } $$

where *r* is the radial position from the center of the rotation, *ρ* is the medium density, *η* is the medium viscosity, and *f* is the rotational frequency. The calculated value of *τ* at 1.0 cm away from the center of rotation was 475.8 dyne/cm^2^ in DMEM and 494.9 dyne/cm^2^ in RPMI. Here, the medium viscosity was considered to be 0.0073 dyne∙s/cm^2^ in DMEM, and 0.0079 dyne∙s/cm^2^ in RPMI [23, 24].

Comparing the optical images captured before and after the spinning, it is evident that the number of cells that remain after spinning is reduced in all cell types (Fig. 4b). Looking at the same position indicated by a square in the images before and after spinning, the loss of cells by spinning is obvious. There was a significant difference between the different cells in medium in the number of remaining cells after the spinning. In the case of MCF10A, a considerable number of cells disappeared, and a small percentage of cells remained after the spinning, while a relatively large number of cells remained for the three types of cancer cells. The fraction of adherent cells after spinning was as follows: MCF10A (59.9%), MCF7 (92.5%), T47D (90.3%), and MDA-MB-231 (87.8%) (Fig. 4c). This result indicates that the chemical adherence of cancer cells is more than twice that of normal cells, and the adhesion of T47D cells is the strongest among the breast cancer cells.

Suppose that the two forces of adhesion and gravity are applied to the cells attached to the substrate (Fig. 4d). The two forces are equal in size and acting on the cells in opposite directions. When the cell rotates, it experiences a centrifugal force directed away from the axis of rotation, and a centripetal force in the inward direction. The centripetal force can be regarded as the adhesive force. Additionally, the shear stress caused by the rotating medium is applied to the cells in the same direction as the centrifugal force. When the equilibrium state between the centrifugal force and adhesive forces breaks down because of the shear stress, the cells detach from the matrix. Therefore, we can estimate the adhesion strength from the number of cells remaining after the spinning.

### Quantitative analysis of single cell size

**Fig. 5:**
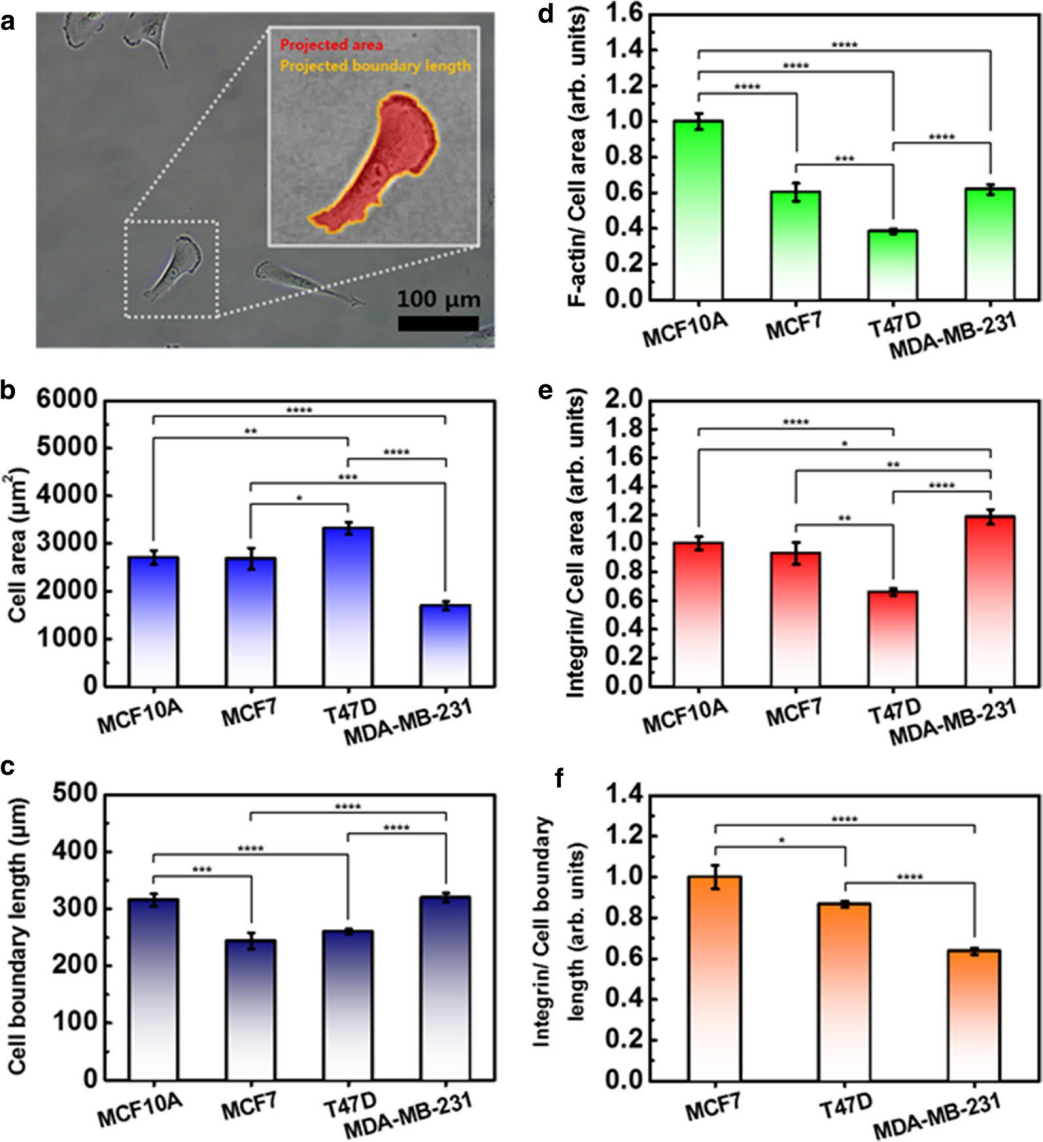
Single cell size quantification for analyzing cell mechanics. **a** Projected area and boundary length of cell was calculated using image processing software (Gwyddion V2.52). **b** The area was determined for 20 cells in each group. **c** Peripheral length around the cell was determined for 20 cells. **d** Amount of F-actin and (**e**) integrin were divided by the cell area. **f** The amount of integrin in three cancer cells was divided by the peripheral length. Each value represents mean ± SEM (*p ≤ 0.05, **p ≤ 0.01, ***p ≤ 0.001, ****p ≤ 0.0001)

Cell-to-cell heterogeneity is observed for many biological properties, shape, and size. Heterogeneity is more pronounced within cell types. Therefore, to quantitatively compare the movement of cells with the content of actin and integrin, the size of the cells should be considered. Using image processing software (Gwyddion V2.52, Czech Metrology Institute, Jihlava, Czech), the projected area and boundary length of the cell was calculated (Fig. 5a). In each group, 20 cells were analyzed, and the values were averaged. T47D revealed the largest area, while MDA-MB-231 was smallest. MCF10A was similar in area to MCF7. The measured cell areas were as follows: MCF10A at 2703 ± 142 µm^2^, MCF7 at 2679 ± 225 µm^2^, T47D at 3317 ± 128 µm^2^, and MDA-MB-231 at 1696 ± 93 µm^2^ (Fig. 5b). The cell boundary was longest in MCF10A, while it was shortest in MCF7. The measured cell boundary length was as follows: MCF10A at 315.3 ± 10.9 µm, MCF7 at 243.8 ± 14.1 µm, T47D at 259.9 ± 4.6 µm, and MDA-MB-231 at 319.6 ± 7.8 µm (Fig. 5c).

For comparison, the contents of F-actin and integrin were divided by the cell area, and then the values were normalized by their value in MCF10A. Here, the contents of F-actin and integrin analyzed were obtained from Figs. 2b and B, respectively. The content of F-actin per area was the largest in MCF10A, followed by MDA-MB-231, MCF7, and T47D (Fig. 5d). Interestingly, this result shows good agreement with the elastic property of the cells mentioned in Fig. 1i. The density of integrin was largest in MDA-MB-231 and smallest in T47D (Fig. 5e). The difference between MCF10A and MCF7 was not significant. Because the integrin was distributed throughout MCF10A, we may assume that the calculated density was almost same within the cells. However, in the case of the cancer cells, most of the integrin was found in the periphery region. Therefore, the integrin content around the nucleus may have been smaller than the calculated value, and that of the periphery region may have been larger than the calculated value. For the three types of cancer cells, the contents of integrin were divided by the boundary length of the cell taking into consideration the distribution region, and then the values were normalized by their value in MCF7 (Fig. 5f). From the viewpoint of the cell boundary, the content of integrin per length was largest in MCF7, followed by T47D and MDA-MB-231. This result shows good agreement with the physical adhesion strength between the cancer cells (Fig. 4c).

## Discussion

Cell movement is a highly dynamic process that is a necessary function in a variety of biological processes such as growing, dividing, wound healing, and cancer metastasis. Cell movement is a complex phenomenon driven by external and internal forces. The external forces, including viscous force or resistance, are generated from the surrounding medium and the cell-substrate interactions. The internal forces are mainly driven by the cytoskeleton. Cells have their own unique migration characteristics, and the speed and direction of migration change due to external factors such as substrate rigidity and viscosity. In this study, we compared migration characteristics between normal breast cells and breast cancer cells, and the difference was interpreted for cellular elasticity and adhesion between the cells and substrate. Because cellular elasticity is mainly regulated by F-actin, the amount and distribution of the F-actin was investigated. Cell adhesion was analyzed by the amount and distribution of integrin.

**Fig. 6:**
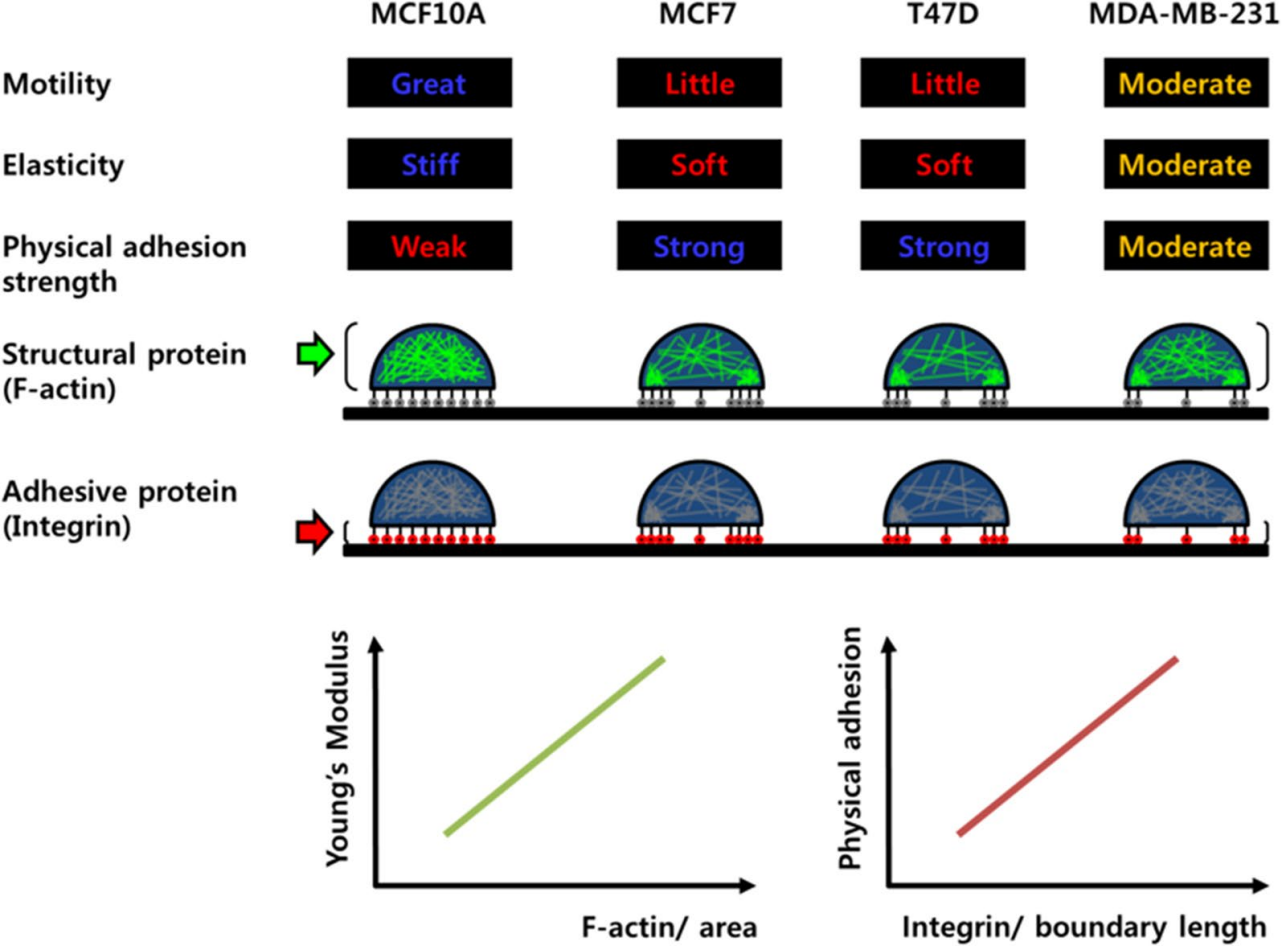
Schematic diagram showing correlation between the motility and mechanical properties. Cell motility shows a strong relation with cellular elasticity and physical adhesion strength. Young’s modulus of the cells shows a linear proportion with F-actin density. Physical adhesion strength was proportional to the integrin amount divided by boundary length

In this work, we demonstrated the correlation of several characteristics between a cell’s motility and its mechanical property (Fig. 6). First, cells with excellent motility are stiff and have weak physical adhesion with the substrate. However, cells with poor motility are soft and firmly physically attached to the substrate. Numerous studies have been carried out on the relationship between cell motility and elasticity in recent years. In particular, because it is known that the elasticity of cancer cells is different from that of normal cells, many efforts have been made to explain the metastasis of cancer cells in relation to their elasticity. However, the correlation between cell motility and elasticity has been a poorly understood and controversial issue until now. At the beginning of this study, it was stated that soft cells have better motility, therefore metastatic cancer cells are soft [9, 25]. In recent years, however, studies contradicting these results have been published [10, 26]. Our results showed that stiff cells are more motile. The normal MCF10A cells were considerably stiffer than the cancer cells and showed excellent motility. The MCF10A cells traveled the longest distance in the 2D movement analysis, and showed the largest migration in terms of number of cells through the membrane pores. In the comparison of cancer cells, the stiffest MDA-MB-231 cells revealed relatively good mobility in both 2D and 3D assays. To migrate, cells continue to form integrin-mediated adhesions with the substrate and to disintegrate. When the adhesion is too strong, the cell migrates slowly because adhesion that is too strong makes the cells immobile [27]. Too-weak adhesion is also not good for migration, due to the insufficient traction force. Therefore, the excellent motility of MCF10A indicated that the cells had sufficient interaction between the integrin and F-actin, even though the adhesion was weaker than in other cancer cells, whereas the migration of cancer cells T47D and MCF7 was disturbed due to excessive adhesion to the substrate.

Second, the stiffness of cells is proportional to the density of F-actin. Actin is a major contributor to cell elasticity. Actin is a highly dynamic protein that undergoes transient synthesis and degradation between G- and F-actin in response to the intracellular environment and external stimuli [28–33]. A permanent change in ratio between G- and F-actin content also occurs due to disease and aging. It is well known that cells become stiff when there is a high amount of F-actin, or when the F-actin forms a bundle or interacts through actin-binding proteins and spreads throughout the cell membrane. MCF10A exhibited this relationship very well. Namely, the cell is stiffest and possesses a large amount of F-actin and well-developed stress fibers. Cancer cells did not exhibit this relationship, presumably because the cell size was quite different and the distribution of F-actin was concentrated only in specific regions of the cell periphery. Thus, if we compare the density of F-actin rather than the total amount considering the different cell sizes, we can see that the density of F-actin is proportional to the cellular elasticity of the cancer cells.

Third, the physical adhesion strength is proportional to the density of integrin, and the chemical adhesion is correlated with the amount of integrin. Cells form contacts with ECM through focal adhesions, which are a type of adhesive contact mediated by transmembrane integrin proteins. One side of the integrin binds ECM glycoproteins such as laminins, collagens, and fibronectin, and the other side binds to F-actin through a physical linkage [10]. Various studies have been carried out to understand the biochemical aspects of integrin-mediated adhesion, and considerable progress has been made. However, the mechanical aspects of adhesion are still poorly understood due to the limitations of quantitative analysis and the complexity of adhesion. Adhesion strength, which is the minimum force needed to detach a single cell from the substrate, is modulated by several factors. Adhesion is strengthened as the cell–substrate contact area increases by cell spreading, clustering by receptors, and focal adhesion assembly through interaction with the cytoskeleton [34]. The adhesion strength increases with the amount of bound integrin [35]. Our result demonstrated that physical adhesion strength is proportional to the density of integrin. As shown in Figs. 3b and 4c, there seems to be no correlation between the amount of integrin and the adhesion strength. However, the amount of integrin divided by the perimeter of the cell shows a linear relationship with physical adhesion strength in cancer cells. This result indicates that the density of integrin provides more reliable information about the mechanical aspects of the adhesion due to the difference in the cell size and distribution of integrin. However, the chemical adhesion between the cells and the substrate seems to be related to the amount of integrin. When the cells were treated with trypsin–EDTA, the normal MAC10A, which has the largest amount of integrin, showed no significant change in morphology for a relatively long time. However, the morphology of the three cancer cells having a relatively small amount of integrin was more easily changed. Definite morphological changes were induced by disrupting the integrin-mediated adhesion.

## Conclusion

In summary, we focused on identifying factors related both qualitatively and quantitatively to cell motility. Both cellular elasticity and physical adhesion to the ECM showed strong correlation with cell motility. The densities of F-actin and integrin exhibited linear correlations with cellular elasticity and adhesion strength, respectively. Therefore, the densities of F-actin and integrin could be used as biomarkers of cell motility.

## Supplementary information

**Additional file 1: Fig. S1.** DAPI nuclear stain of a normal cell (MCF10A) and breast cancer cells (MCF MCF7, T47D, MDA-MB-231). Blue is DAPI nuclear stain, which shows that there is only one nucleus per cell. **Fig. S2.** Linear regression analysis between (A) Young’s modulus and F-actin/area, and (B) physical adhesion and Integrin/boundary length. A high correlation can be found in adjusted R-squared of 0.92167 and 0.97347 for the relations, respectively.

## Data Availability

The datasets used and/or analyzed during the current study are available from the corresponding author on reasonable request.

## Abbreviations

ECM: Extracellular matrix
AFM: Atomic force microscopy
SCTS: Single cell tracking system
G-actin: Free monomer
F-actin: Microfilament
LatA: Latrunculin A

## References

[1] Alberts B, Johnson A, Lewis J, Raff M, Roberts K, Roberts K, Walter P (2002). Molecular biology of the cell.

[2] Colin DP, Panagiotis M, Konstantinos K (2017). Cancer cell motility: lessons from migration in confined spaces. Nat Rev Cancer.

[3] Ananthakrishnan R, Ehrlicher A (2007). The forces behind cell movement. Int J Biol Sci..

[4] Murphy-Ullrich JE (2001). The de-adhesive activity of matricellular proteins: is intermediate cell adhesion an adaptive state?. J Clin Invest..

[5] Lindberg U, Karlsson R, Lassing I, Schutt CE, Höglund AS (2008). The microfilament system and malignancy. Semin Cancer Biol.

[6] De La Cruz EM, Gardel ML (2015). Actin mechanics and fragmentation. J Biol Chem.

[7] Wagh AA, Roan E, Chapman KE, Desai LP, Rendon DA, Eckstein EC, Waters CM (2008). Localized elasticity measured in epithelial cells migrating at a wound edge using atomic force microscopy. Am J Physiol Lung Cell Mol Physiol.

[8] Krause M, Wolf K (2015). Cancer cell migration in 3D tissue: negotiating space by proteolysis and nuclear deformability. Cell Adh Migr..

[9] Wenwei X, Roman M, Byungkyu K, Lijuan W, John M, Todd S (2012). Cell stiffness is a biomarker of the metastatic potential of ovarian cancer cells. PLoS ONE.

[10] Messica Y, Laser-Azogui A, Volberg T, Elisha Y, Lysakovskaia K, Eils R, Gladilin E, Geiger B, Beck R (2017). The role of vmentin in regulating cell invasive migration in dense cultures of breast carcinoma cells. Nano Lett.

[11] Khalili AA, Ahmad MR (2015). A review of cell adhesion studies for biomedical and biological applications. Int J Mol Sci.

[12] Spangenberg C, Lausch EU, Trost TM, Prawitt D, May A, Keppler R, Fees SA, Reutzel D, Bell C, Schmitt S, Schiffer IB, Weber A, Brenner W, Hermes M, Sahin U, Türeci O, Koelbl H, Hengstler JG, Zabel BU (2006). ERBB2-mediated transcriptional up-regulation of the α5β1 integrin fibronectin receptor promotes tumor cell survival under adverse conditions. Cancer Res.

[13] Zou JX, Liu Y, Pasquale EB, Ruoslahti E (2002). Activated Src oncogene phosphorylates R-ras and suppresses integrin activity. J Biol Chem.

[14] Fuhrmann A, Banisadr A, Beri P, Tlsty TD, Engler AJ (2017). Metastatic state of cancer cells may be indicated by adhesion strength. Biophys J.

[15] Cappella B, Dietler G (1993). Force-distance curves by atomic force microscopy. Surf Sci Rep.

[16] James ENJ, Judith AC, Feng X, Miria EB, Jennifer EA, Katarzyna MS, Pina C (2014). An introduction to the wound healing assay using live-cell microscopy. Cell Adh Migr..

[17] Bise R, Kanade T, Yin Z, Huh S. Automatic cell tracking applied to analysis of cell migration in wound healing assay. Conf Proc IEEE Eng Med Biol Soc. 2011;6174–6179. 10.1109/IEMBS.2011.6091525 22255749

[18] Michael BD, Amil AJ, Ambalangodage CJ (2015). Current wound healing procedures and potential care. Mater Sci Eng C Mater Biol..

[19] Grada A, Otero-Vinas M, Prieto-Castrillo F, Obagi Z, Falanga V (2016). Research techniques made simple: analysis of collective cell migration using the wound healing assay. J. Invest. Dermatol..

[20] Li M, Liu L, Xi N, Wang Y, Dong Z, Xiao X, Zhang W (2011). Atomic force microscopy imaging and mechanical properties measurement of red blood cells and aggressive cancer cells. Sci China Life Sci..

[21] Binnig G, Quate CF, Gerber C (1986). Atomic force microscope. Phys Rev Lett.

[22] Amelia AK, Mohd RA (2015). A review of cell adhesion studies for biomedical and biological applications. Int J Mol Sci.

[23] Rinker KD, Prabhakar V, Truskey GA (2001). Effect of contact time and force on monocyte adhesion to vascular endothelium. Biophys J.

[24] Ahmad KR, Kumar A, Sadouki F, Lorenz C, Forbes B, Dailey LA, Collins H (2012). The delivered dose: applying particokinetics to in vitro investigations of nanoparticle internalization by macrophages. J Control Release.

[25] Lautscham LA, Kämmerer C, Lange JR, Kolb T, Mark C, Schilling A, Strissel PL, Strick R, Gluth C, Rowat AC, Metzner C, Fabry B (2015). Migration in confined 3D environments is determined by a combination of adhesiveness, nuclear volume, contractility, and cell stiffness. Biophys J.

[26] Esra R, Kristina RW, Christopher MW (2013). Cell stiffness and the onset of epithelial cell migration in wound repair. Am J Respir Crit Care Med.

[27] Gupton SL, Waterman-Storer CM (2006). Spatiotemporal feedback between actomyosin and focal-adhesion systems optimizes rapid cell migration. Cell.

[28] Daniel AF, Dyche RM (2010). Cell mechanics and the cytoskeleton. Nature.

[29] Lambrechts A, van Troys M, Ampe C (2004). The actin cytoskeleton in normal and pathological cell motility. Int J Biochem Cell Biol.

[30] Olson MF, Sahai E (2009). The actin cytoskeleton in cancer cell motility. Clin Exp Metastasis.

[31] Yamazaki D, Kurisu S, Takenawa T (2005). Regulation of cancer cell motility through actin reorganization. Cancer Sci.

[32] Yamaguchi H, Condeelis J (2007). Regulation of the actin cytoskeleton in cancer cell migration and invasion. Biochim Biophys Acta.

[33] Vignjevic D, Montagnac G (2008). Reorganisation of the dendritic actin network during cancer cell migration and invasion. Semin Cancer Biol.

[34] Palecek SP, Loftus JC, Ginsberg MH, Lauffenburger DA, Horwitz AF (1997). Integrin-ligand binding properties govern cell migration speed through cell-substratum adhesiveness. Nature.

[35] Gallant ND, Michael KE, García AJ (2005). Cell adhesion strengthening: contributions of adhesive area, integrin binding, and focal adhesion assembly. Mol Biol Cell.

