# Supplementary material for: Biomarkers to quantify cell migration characteristics
Source: Cancer Cell Int. 2020 Jun 5;20:217. doi: 10.1186/s12935-020-01312-w (PMC7275471; doi:10.1186/s12935-020-01312-w)

Supporting material

Fig. S1. DAPI nuclear stain of a normal cell (MCF10A) and breast cancer cells (MCF MCF7, T47D, MDA-MB-231). Blue is DAPI nuclear stain, which shows that there is only one nucleus per cell.

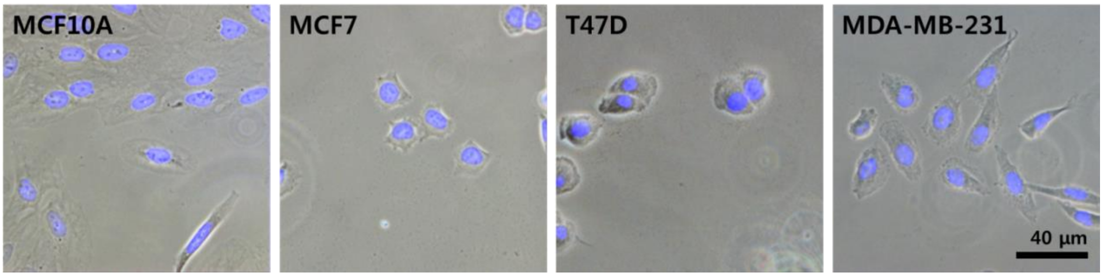

Fig. S2. Linear regression analysis between (A) Young's modulus and F-actin/area, and (B) physical adhesion and Integrin/boundary length. A high correlation can be found in adjusted R-squared of 0.92167 and 0.97347 for the relations, respectively.

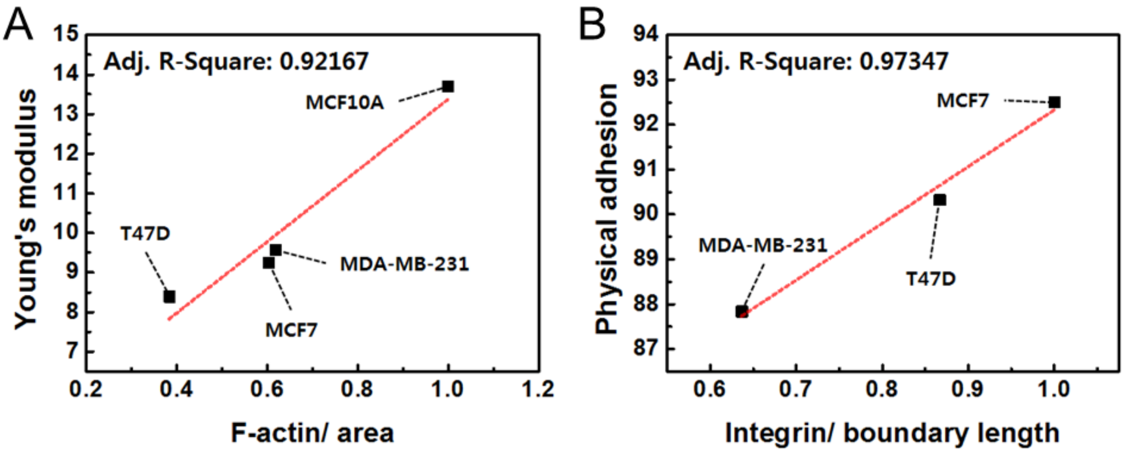

Supplement: Supplementary file 1 — Additional file 1: Fig. S1. DAPI nuclear stain of a normal cell (MCF10A) and breast cancer cells (MCF MCF7, T47D, MDA-MB-231). Blue is DAPI nuclear stain, which shows that there is only one nucleus per cell. Fig. S2. Linear regression analysis between (A) Young’s modulus and F-actin/area, and (B) physical adhesion and Integrin/boundary length. A high correlation can be found in adjusted R-squared of 0.92167 and 0.97347 for the relations, respectively. [file 12935_2020_1312_MOESM1_ESM.pdf]
